# Editorial for the Special Issue: Pathophysiology of Chronic Kidney Disease and Its Complications

**DOI:** 10.3390/biomedicines12020416

**Published:** 2024-02-10

**Authors:** Yuji Oe

**Affiliations:** Division of Nephrology, Rheumatology and Endocrinology, Tohoku University Graduate School of Medicine, Sendai 980-8574, Japan; yuji-oe@med.tohoku.ac.jp

Chronic kidney disease (CKD) is a risk factor for end-stage kidney disease, requiring renal replacement therapy. Additionally, CKD is linked to various complications, including cardiovascular disease, bone abnormalities, muscle atrophy, and cognitive dysfunction, all of which detrimentally impact the prognosis and the quality of life of CKD patients [1,2]. This Special Issue spotlights recent findings regarding the pathogenesis of CKD and its associated complications. In this editorial, the editor briefly outlines the contributing studies, which will enhance our understanding of CKD pathophysiology and its treatment.

This Special Issue contains several reviews of the pathogenesis and complications of CKD. Frak et al. outlined the latest information on the pathophysiology of CKD, including oxidative stress, inflammation, neutrophil gelatinase-related lipocalin, matrix metalloproteinases, and uremic toxins. The potential application of new molecularly targeted agents for interleukin 6 and transforming growth factor-β signaling to CKD was also discussed [3]. Stroke occurs more frequently in patients with CKD. A low eGFR and albuminuria are known risk factors for stroke [4]. Kourtidou et al. reviewed the various causes of the high prevalence of stroke in CKD patients [5]. Stroke risk factors, such as atrial fibrillation, hypertension, and carotid atherosclerosis, are reported to be more frequent in patients with CKD. Additionally, uremic toxins accumulate in CKD patients, exacerbating this risk. Oe et al. reviewed the risk of thrombosis due to the activation of coagulation factor III (tissue factor) and the mechanism of renal damage caused by coagulation proteases [6]. They focused on the link between gut microbiota, urea toxins, and coagulation abnormalities in CKD patients. Obesity is considered an established risk factor for CKD [7]. Kreiner et al. reviewed the current understanding of obesity-related kidney disease pathogenesis and discussed non-pharmacological and pharmacological options, including SGLT2 inhibitors, non-steroidal mineralocorticoid receptor antagonists, and GLP1 analogs [8].

Several research articles have also been published in this Special Issue. Patients with cardiorenal syndrome (CRS), where acute or chronic dysfunction of the heart or kidneys leads to the dysfunction of the other [9], exhibited altered circulating immune cell subset profiles [10]. Duni et al. investigated differences in circulating immune cells between patients with type 2 CRS and those with CKD without cardiovascular disease [11]. They discovered that CD4+ T lymphocytes independently predicted fatal cardiovascular events in their cohort. CKD complicated by heart failure may involve distinct immune mechanisms in its pathogenesis and chronic clinical course compared to CKD without heart failure.

Sarcopenia is one of the CKD complications associated with morbidity and mortality [12]. In CKD patients, various pathologies, including chronic inflammation, oxidative stress, and the accumulation of uremic toxins, may contribute to the risk of developing skeletal muscle abnormalities, such as sarcopenia [13]. Advanced glycation end products (AGEs) are compounds formed through non-enzymatic reactions of reducing sugars and related metabolites with proteins and amino acids. Additionally, AGEs have been demonstrated to bind to the membrane receptor RAGE, causing oxidative stress and inflammation in CKD patients [14]. In a study conducted by Molinari et al., 117 patients with CKD were examined, revealing that AGEs, but not soluble RAGEs, were associated with the presence of sarcopenia in older CKD patients [15]. Therefore, AGEs may contribute to the intricate pathophysiology leading to sarcopenia development in patients with CKD.

Changes in plasma protein profiles during various stages of CKD are crucial for identifying early diagnostic markers and potential therapeutic targets. To address this, Grgurevic et al. conducted a comprehensive plasma proteome analysis across different CKD stages, resulting in the generation of distinct protein profiles [16]. In total, 453 proteins were identified across all study groups. The findings revealed that pivotal events influencing the pathogenesis and progression of the disease were most pronounced in CKD stage 2. These events specifically centered around inflammation, lipoprotein metabolism, angiogenesis, and tissue regeneration. The researchers hypothesized that CKD stage 2 represents a critical tipping point in the disease’s progression, highlighting its significance for potential therapeutic interventions.

Makino et al. analyzed the prognostic impact of nutritional and inflammatory status on non-metastatic renal cell carcinoma. In their analysis of 213 Japanese patients, they examined how several inflammatory and nutritional indices were associated with overall survival [17]. They found that the index neutrophil-to-lymphocyte ratio and C-reactive protein-to-albumin ratio independently contributed to prognostic factors.

Basic research is also included in this Special Issue. Recently, a new class of antihyperglycemic drugs, sodium-glucose co-transporter 2 (SGLT2) inhibitors, has shown promise in protecting the kidneys and heart of patients with CKD, both with and without diabetes [18,19]. Alongside the focus on SGLT2, interest in SGLT1 as a therapeutic target is increasing. In addition to its role in glucose reabsorption in the intestinal tract, SGLT1 is expressed in the macula densa and plays a role in glomerular hemodynamics and blood pressure control [20,21]. Matthew et al. researched the regulation of SGLT1 expression by the sympathetic nervous system (SNS) [22]. By measuring SGLT1 expression in the kidneys of neurogenic hypertensive mice and treating human proximal tubular cells with norepinephrine, they demonstrated that SNS activation upregulates SGLT1 expression. Consequently, inhibiting SGLT1 may be a valuable therapeutic strategy for conditions characterized by increased SNS activity, such as CKD.
